# Prognostic Value of Circadian Rhythm of Brain Temperature in Traumatic Brain Injury

**DOI:** 10.3390/jpm11070620

**Published:** 2021-06-30

**Authors:** Lu-Ting Kuo, Hsueh-Yi Lu, Abel Po-Hao Huang

**Affiliations:** 1 Division of Neurosurgery, Department of Surgery, National Taiwan University Hospital, No. 7 Chung San South Road, Taipei 100, Taiwan; kuoluting@gmail.com; 2 Department of Industrial Engineering and Management, National Yunlin University of Science and Technology, Yunlin 64002, Taiwan; hylu@yuntech.edu.tw; 3 Institute of Polymer Science and Engineering, National Taiwan University, Taipei 100, Taiwan

**Keywords:** organ temperature, traumatic brain injury, postoperative care, hypothermia, circadian rhythm, prognosis

## Abstract

Hypothermia has been used in postoperative management of traumatic brain injury (TBI); however, the rhythmic variation and prognostic value of brain temperature after TBI have never been studied. This study describes diurnal brain temperature patterns in comatose patients with TBI. Mesors of brain temperature, amplitude, and acrophase were estimated from recorded temperature measurements using cosinor analysis. The association of these patterns with clinical parameters, mortality, and functional outcomes in a 12-month follow-up was examined. According to the cosinor analysis, 59.3% of patients presented with circadian rhythms of brain temperature in the first 72 h postoperatively. The rhythm-adjusted mesor of brain temperature was 37.39 ± 1.21 °C, with a diminished mean amplitude of 0.28 (±0.25) °C; a shift of temperature acrophase was also observed. Multivariate logistic regression analysis revealed that initial Glasgow coma scale score, age, elevated blood glucose level, and circadian rhythm of brain temperature seemed to be predictive and prognostic factors of patients’ functional outcomes. For the prediction of survival status, younger patients or those patients with mesor within the middle 50% of brain temperature were more likely to survive. The analysis of brain temperature rhythms in patients with moderate and severe TBI provided additional predictive information related to mortality and functional outcomes.

## 1. Introduction

Improvements in diagnosis, surgical intervention, and intensive care have allowed many patients to survive traumatic brain injury (TBI); however, the long-lasting disability has led to significant functional, emotional, and economic sequelae [1,2]. Long-term outcomes vary greatly, especially in patients with moderate or severe TBI requiring surgery [1,3]. Previous studies have identified clinical parameters to predict conscious and functional recovery, including Glasgow coma scale (GCS) score, age, electroencephalogram (EEG) results, image findings, pupillary response to light, and somatosensory evoked potential (SSEP) [1,3,4,5]. Only age and GCS score were consistently recognized as useful outcome predictors in several studies. EEG has been widely used to evaluate comatose patients; however, its application as a predictor in traumatic coma was not as sensitive as in hypoxic-ischemic coma [6]. Outcomes of patients with TBI are still often unpredictable during the early stage of treatment.

Circadian rhythms are a biological adaptation to the light-dark cycle. They regulate sleep-wake cycles, melatonin levels, body temperature, and other body functions. As a part of the circadian rhythm, brain temperature mostly reflects body temperature under physiological conditions. From human and animal data, brain temperature is generally higher than body temperature by approximately 0.3 °C, and correlates well with body temperature [7,8,9]. However, brain temperature cannot be predicted reliably from core body temperature after TBI [10]. Therapeutic hypothermia (TH) appears to be an efficacious treatment in experimental animals of TBI; however, the benefits of TH remain controversial in patients with TBI [11,12,13,14]. Previous studies have demonstrated the effects of rhythms in blood pressure and heart rate on intracerebral hemorrhage [15] and the effects of rhythms in brain temperature on subarachnoid hemorrhage [16]; however, no studies have examined the effects of brain temperature rhythm in patients with TBI [17]. Therefore, we aimed to characterize the brain temperature rhythm and investigate its prognostic value in terms of postoperative mortality and functional outcomes in patients with moderate to severe TBI.

## 2. Materials and Methods

### 2.1. Study Design

In this prospective, observational study, we collected the clinical characteristics, brain temperature, and postoperative outcomes of patients with TBI. All patients were followed-up after surgery until they regained a Glasgow Outcome Scale (GOS) score of 5 for 12 months. The primary endpoints were mortality and functional outcome following TBI surgery. The study was conducted at the National Taiwan University Hospital according to the applicable local regulations and the Declaration of Helsinki. This study was approved by the institutional review board of the National Taiwan University Hospital (IRB number: 201211025RIB). Written informed consent was obtained from the caregivers of patients, as patients were in the comatose state.

### 2.2. Medical Procedure and Assessments

Upon admission to the hospital, a physician immediately evaluated the GCS score and conducted brain computed tomography (CT). Prehospital management was completed according to the standards of the Taiwan Society of Emergency Medicine, which is compatible with the Brain Trauma Foundation Guidelines for Prehospital Management of Traumatic Brain Injury. At the emergency room (ER), patients were examined and treated following the Advanced Trauma Life Support guidelines and the American Association of Neurological Surgeons/Congress of Neurological Surgeons Guidelines for the Management of Head Injury.

Surgical approach selection (i.e., craniectomy, craniotomy, and/or hematoma evacuation with the intraparenchymal placement of Camino fiberoptic intracranial pressure monitor Model 110-4BT [Camino Laboratories, San Diego, CA 90011, USA]) for an acute subdural hematoma, epidural hematoma, or other intracranial hemorrhages was based on GCS score, pupillary examinations, comorbidities, CT findings, age, and occurrence of neurological deterioration (e.g., decreased GCS score or abnormal pupillary response to light).

All patients were admitted to the intensive care unit (ICU) after surgery. Brain temperature was continuously recorded using an intracranial pressure (ICP) monitoring device screwed into the skull, with hourly data collected for circadian rhythm analysis. Conduction cooling, including ice packs, ice pillow, or circulating cold water blankets, was used for patients with febrile episodes (>38.5 °C). Standard monitoring procedure included invasive measurement of arterial blood pressure (zero-point at the fourth intercostal space at the midclavicular line), pulse oximeter oxygen saturation, end-tidal CO_2_ concentration, and postoperative blood sugar levels. Tympanic body temperature was measured every 3 h, while neurological assessments (e.g., pupil reflex to light) were performed hourly. Mean arterial blood pressure (MAP) and ICP were continuously monitored to maintain a minimum cerebral perfusion pressure (CPP) of 70 mmHg. Blood pressure support (i.e., dopamine and norepinephrine) was provided as necessary.

Postoperative management included ventilation, oxygenation, head elevation (30°), fluid resuscitation, and the use of sedatives (i.e., short-acting benzodiazepines). Hypothermia treatment was not applied, while antibiotic prophylaxis was administered to each patient for at least 3 days after surgery. Enteral feeding was initiated on postoperative day 2 using nasogastric or orogastric tubes for patients without upper gastrointestinal hemorrhage. An ICP of >25 mmHg for 4 h during the first 3 days was considered the threshold to initiate head CT scan and treatment; otherwise, the head CT scan was performed on postoperative day 3.

After being transferred to the ward, patients participated in outpatient rehabilitation programs and were followed-up for at least 12 months. Postoperative follow-up included GOS assessment and survival status. The GOS is a 5-point scale: 1, death; 2, vegetative state; 3, severe disability; 4, moderate disability; and 5, good recovery. A favorable outcome was defined as a GOS of >3 at 12 months, and an unfavorable outcome was defined as a GOS of 1–3 at 12 months.

### 2.3. Population

All patients were admitted to the National Taiwan University Hospital–Yunlin branch from May 2011 to December 2017. Patients who met the following criteria were included: moderate to severe TBI (GCS score 3–12 at admission), >18 years of age, underwent TBI surgery with ICP monitoring, and brain temperature recorded for at least 72 h after surgery. TBI was defined when a patient had at least one of the following diagnoses: subdural hematoma, epidural hematoma, cerebral contusion, subarachnoid hemorrhage, or diffuse axonal injury. Patients with pre-existing brain diseases (e.g., brain tumor, stroke, meningitis), substance abuse, combined traumatic injuries (rib fracture, hemothorax, liver or spleen laceration, or bone fracture except skull bone), brainstem injury, diffuse axonal injury, infection within 72 h after admission, or survival time less than 3 days after TBI surgery were excluded. Patients who signed a do-not-resuscitate order were also excluded.

### 2.4. Brain Temperature Analysis

Temperature measurements over 72 h were divided into 24-h segments, beginning 2 h postoperatively, to ensure sufficient temperature records for unbiased estimation of cosinor parameters and to minimize potential confounders from postoperative hypothermia.

The following brain temperature characteristics were collected: waveform of temperature oscillations; mean level (referred to as the mesor) of temperature oscillations; amplitude of the temperature rhythm, defined as half the difference between the lowest and highest temperature; and the acrophase, the time when the temperature rhythm reached its maximum value for the day. The mesor is the circadian rhythm-adjusted mean based on the parameters of a cosine function. The mesor of the body temperature varies depending on where the body temperature is measured.

The circadian rhythm of brain temperature was analyzed using the cosinor method, which is the most used statistical method for modelling diurnal temperature patterns. Cosinor analysis involves fitting the data to a 24-h cosine curve, with estimates of the rhythm parameters, including mesor, amplitude, and acrophase [18].

### 2.5. Measurement of Light Intensity in an ICU

**Figure 1:**
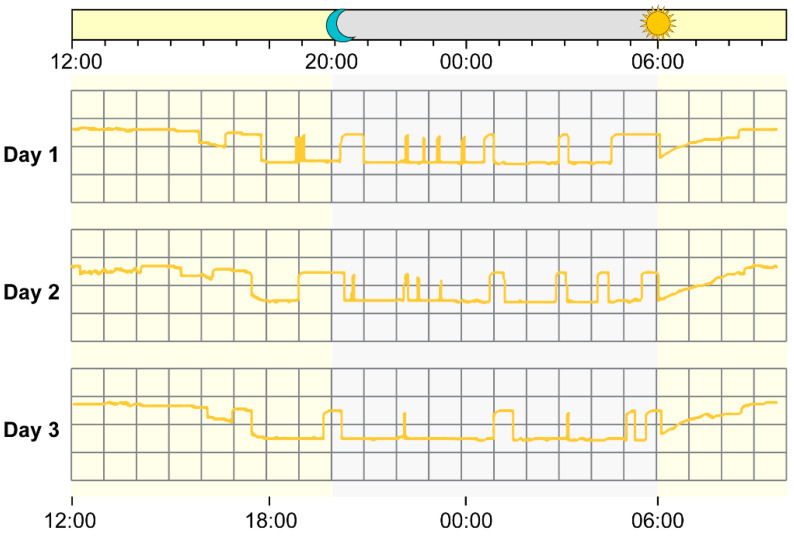
Light pattern in the intensive care unit.

The ICU had natural light through translucent frosted glass windows and fluorescent lighting turned on from 06:00 to 22:00. To evaluate the effect of light intensity in the ICU, illumination was recorded using the Actiwatch-L monitor (MiniMitter-Respironics Co., Inc., Bend, OR 97331, USA). The monitor was placed <100 cm from the head of the patient; light intensity was measured for 7 days with an acquisition interval of 30 s. Light intensity was also analyzed using the cosinor analysis to acquire the 24-h pattern. According to the Actiwatch-L monitor, the light pattern in the ICU showed diurnal changes and was generally brighter during the daytime (Figure 1).

### 2.6. Statistical Analyses

A chi-squared test (or Fisher’s exact test) and t-test were used to compare outcomes between patient subgroups for categorical data and continuous data, respectively. Significant variables found in the univariate analysis were added into multivariate logistic regression models, which were performed with stepwise selection to identify independent predictors of mortality and functional outcomes. Missing data were not included in the analysis. Statistical significance was set at a *p*-value of <0.05. Data were analyzed using SPSS 14.0 for Windows (SPSS, Inc., Chicago, IL 60601, USA).

## 3. Results

### 3.1. Demographic Data

The demographics and clinical characteristics of patients are presented in Table 1. Overall, 108 patients were enrolled who met the study criteria. The majority were men (68.5%) who had TBI from motor vehicle accidents (80.6%). The mean age ± standard deviation (SD) was 52.3 ± 20.7 years (range: 18–80 years). Fifty-seven patients (52.8%) had subdural hematoma, 15 (13.9%) had epidural hematoma, 15 (13.9%) had contusional intracerebral hemorrhage (ICH), and 19 (19.4%) had other diagnoses, such as arachnoid hemorrhage or a combination of two or more diagnoses. The mean GCS score ± SD at admission was 6.9 ± 2.8, with 31 patients (28.7%) having moderate TBI (GCS: 9–12) and 77 (71.3%) having severe TBI (GCS: 3–8). Among the 108 TBI cases, the main diagnosis for surgical treatment was subdural hematoma in 74 cases, epidural hematoma in 13 cases, and traumatic contusion in 21 cases. The surgical approach for these TBI patients included ICP monitor insertion in 8 patients, craniectomy with ICP monitor insertion in 68 patients, and craniotomy for hematoma evacuation with ICP monitor insertion in 32 patients.

### 3.2. Postoperative Outcome

**Figure 2:**
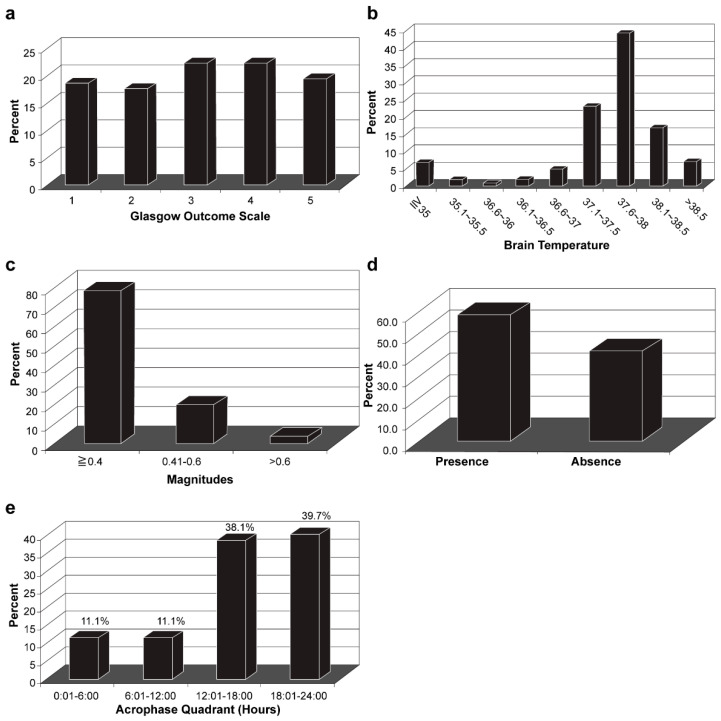
Analysis of brain temperature and functional outcome at 12 months: (**a**) Glasgow Outcome Scale score 12 months after surgery; (**b**) Mesor of brain temperature; (**c**) Amplitude of brain temperature; (**d**) Presence of circadian rhythm of brain temperature; (**e**) Acrophase of brain temperature.

After surgery (Table 1), the mean ICP ± SD in the first 24 h was 16.4 ± 14.8 mmHg, with 26.9% of patients having an ICP > 20 mmHg. The mean CPP ± SD in the first 24 h was 78.5 ± 15.0 mmHg. At 12 months post-surgery, 45 patients (41.7%) showed favorable outcomes (GOS > 3) and 20 patients (18.5%) had died (Figure 2a).

### 3.3. Characteristics of Brain Temperature Analysis

The mesor ± SD was 37.4 ± 1.2 °C, with 21.30% of patients showing 37.1 °C–37.5 °C, 40.7% showing 37.6 °C–38.0 °C, and 15.7% showing 38.1 °C–38.5 °C (Figure 2b). The amplitude of brain temperature for most patients (82.4%) was <0.4 °C (Figure 2c). According to the cosinor analysis, 63 patients (58.3%) showed circadian rhythm (Figure 2d), defined as R^2^ ≥ 0.10. Regarding the acrophase of brain temperature in 63 patients with intact circadian rhythm, the acrophase of 24 patients (38.1%) fell between 12:00 and 18:00, and the acrophase of 25 patients (39.7%) peaked between 18:00 and 24:00 (Figure 2e). Forty-nine of the 63 patients (77.7%) showed a decreased amplitude of brain temperature.

### 3.4. Analysis of Predictors for Postoperative Outcome

The univariate analysis of postoperative outcomes revealed that sex, age, diabetes, hypertension, initial GCS score, glucose level, and mesor and rhythm of brain temperature might be prognostic factors of postoperative functional outcomes (Table 2). Patients with favorable outcomes (GOS > 3) at 12 months were mostly men, significantly younger, had higher GCS scores, and lower glucose levels before surgery; there was a lower proportion of patients with diabetes or hypertension than of patients with unfavorable outcomes (GOS = 1–3) at 12 months. Additionally, the cohort with favorable outcomes showed a higher mesor in the first 72 h after surgery and had a higher proportion of patients with brain temperature rhythm. Moreover, age, initial GCS score, ICP and CCP 24 h after surgery, and mesor of brain temperature seemed to be prognostic factors of survival status. Patients alive at 12 months were significantly younger, had higher GCS scores before surgery, had higher CPP in the first 24 h after surgery, had lower ICP in the first 24 h after surgery, and showed higher mesor than patients who had died.

The differences between male and female patients for all parameters were tested separately in the unfavorable, favorable, dead, and alive outcomes. The parameters with significant differences between male and female patients are shown in Table 3. Female patients with unfavorable outcomes had a significantly higher proportion of diabetes mellitus (34.6%) than male patients (13.5%); female patients with favorable outcomes had a significantly higher proportion of elevated glucose levels (87.5% > 120 mg/dL), and all their mesors were within the middle 50% of brain temperature; female patients who survived at 12 months after surgery had significantly higher average age (57.7 years), higher proportion of diabetes mellitus (25.9%), higher proportion of elevated glucose levels (88.9% > 120 mg/dL), and lower ICP (3.7% > 20 mmHg). No significant difference was found among sex in the dead outcome group.

Potential predictors identified in the univariate analysis were included in the multivariate analysis (Table 4). The functional outcome 12 months after surgery was significantly associated with age, elevated blood glucose level, initial GCS score, and the presence of brain temperature rhythm. Specifically, patients with brain temperature rhythms were 5.28 times more likely to have a favorable functional outcome 12 months after the surgery (odds ratio [OR] 5.28; 95% confidence interval [CI] 1.61–17.64). Older patients were less likely to have favorable outcomes (OR 0.94; 95% CI 0.90–0.97). Patients with a higher GCS score before surgery were more likely to have a favorable outcome (OR 1.53; 95% CI 1.21–1.93). Patients with initial blood glucose of >120 mg/dL were 0.21 times less likely to have a favorable outcome (OR 0.21; 95% CI 0.06–0.76). The survival status 12 months after surgery was significantly associated with mesor and age. Results revealed that older patients were less likely to survive after surgery (OR 0.96; 95% CI 0.93–1.00). When the mesor was within the middle 50% of brain temperature, patients were 4.77 times (OR 4.77; 95% CI 1.40–16.21) more likely to survive.

## 4. Discussion

Our study provides a comprehensive characterization and analysis of brain temperature rhythm in patients who underwent surgery for moderate to severe TBI and examined the association of brain temperature rhythm with mortality and 12-month functional outcomes. The results of the multivariate analysis demonstrated that age, initial GCS score, blood glucose level, and the presence of brain temperature rhythm were significantly correlated with the functional outcome 12 months after surgery, while age and mesor of brain temperature were associated with patient survival. However, ICP and CPP were not independent predictors of postoperative outcomes in our cohort, which is supported by the findings of other well-designed studies [19,20,21].

Our results demonstrated that patients with mesor of brain temperature within the middle 50% during the first 3 days after surgery had a better chance of survival. This is comparable with the findings of previous studies that suggested that patients who had the highest and lowest average brain temperatures after TBI were more likely to have poor outcomes [22,23]. Variation in brain temperature is mainly dependent on cerebral blood flow, blood temperature, and cerebral metabolism. The change in brain temperature was found to be significantly correlated with the occurrence of febrile episodes and increased ICP [24]. The elevation of brain temperature may signify the reset of the hypothalamic thermoregulatory center, posttraumatic changes in the brain metabolism, hyperemia, or a local inflammatory response [25,26,27]. In contrast, a decrease in brain temperature may reflect a decrease in cerebral blood flow owing to increased ICP. Brain temperature is normally higher than the body core temperature by 0.3 °C, with variability [10,28]. Studies have reported that inversion of the brain/body temperature gradient is associated with poor outcome in severe TBI [23,29]. In extreme cases of acute hypothalamic instability after TBI, both severe hypothermia and may occur [30,31], and these may be life-threatening conditions if not judiciously managed in time.

In addition to the absolute value of temperature, circadian rhythms are another feature of brain and body temperatures. The rhythm is controlled by a central pacemaker in the suprachiasmatic nuclei (SCN), which is situated in the anterior part of the hypothalamus. The cosinor analysis in our study identified changes in brain temperature rhythm after TBI. The brain temperature rhythm was abnormal in 41.7% of patients with TBI. In 58.3% of patients with preserved brain temperature rhythm, amplitudes of brain temperature were blunted in 82.4% of patients, and acrophases shifted in 61.9% of patients.

Impaired rhythm of brain temperature after TBI may be explained by several factors, such as direct or indirect injury to the hypothalamus, medical care, nursing activities, and ICU settings. Previous studies have revealed TBI may cause microscopic hemorrhage of the retinohypothalamic tract—the neuronal pathway extending from the SCN to the pineal gland that synthesizes melatonin, which may lead to the disruption of circadian rhythms [32,33]. Increased ICP after TBI is considered the main possible mechanism leading to SCN injury, compression of hypothalamic structures, and impairment of endogenous regulatory mechanisms, especially in cases with large hematoma or subsequent brain edema. Moreover, increased ICP and stress have been found to cause apoptosis in the hypothalamus and pituitary gland, which may explain hypothalamic pituitary dysfunction after TBI [34,35,36]. Similarly, in an autopsy report, hypothalamic lesions with microhemorrhage or ischemic necrosis were found in 42.5% of 106 patients who died after severe TBI [37]. The presence or absence of the circadian rhythm of brain temperature indicates whether profound neuronal damage is present; the circadian rhythm of brain temperature may affect the outcome, particularly regarding damage in the hypothalamus. Our results further suggest that the presence of the brain temperature rhythm may be used to predict long-term functional outcomes. Gleason et al. studied 26 patients with TBI and subarachnoid hemorrhage (SAH) and found similar results; patients with favorable outcomes showed improved circadian rhythmicity and entrainment [38]. Rzechorzek et al. studied 40 TBI patients and found that older TBI patients lacking circadian rhythmicity are at the greatest risk of death [39].

Considering multiple factors may contribute to the disturbance of brain temperature rhythm and the shift of acrophase, further study is needed to determine other potential factors. Our results could potentially be applied to other neurocritical patients, such as patients with SAH and intracerebral hemorrhage [38]. In addition, circadian rhythm sleep-wake disorders have been identified as an important and addressable issue in mild TBI and post-concussion patients [40,41]. Since non-surgical brain temperature measurement with MRI is feasible [41], further application to this group of patients is also promising.

Our study had several limitations. First, the heterogeneity of patients’ characteristics, including age, comorbidity, and the type of hematoma (location, depth, volume, etc.) may affect the postoperative outcome. The mean age of our patients (57 years) was greater than that of the populations of most TBI studies because the data were collected in Yunlin County, Taiwan, where 13% of the population is over 65 years of age. The effects of age on the pattern of circadian rhythms, such as decreased magnitude, lower mean values, and greater intra- and inter-individual differences, should be considered. Notably, a relatively stable rhythm of body temperature was observed in old age until the end of life [42,43]. Second, the study analyzed clinical data in a single hospital with a small population; therefore, the study results lack generalizability, and further large-scale investigation is warranted. Third, as with all studies focusing on moderate and severe TBI, the initial GCS sum score as a prognostic marker has its limitation, since it usually fluctuates significantly over time. The GCS sum score is not an ordinal scale regarding the outcome after TBI, particularly not at the lower end of the scale representing coma (GCS 3–7) [44,45]. Lastly, our results may be biased owing to the lack of control and differences in clinical decisions/experiences among neurosurgeons and neurocritical care experts. Despite the above limitations, identifying the predictive value of brain temperature has clinical implications in the management of TBI, especially for the decision-making process involving communication between neurosurgeons, neurocritical care experts, and the patient’s family [46]. Early prediction of outcomes may allow clinicians to recognize futility in the early postoperative course and facilitate communication for achieving optimal management to avoid deleterious effects on the patient, family, and healthcare system.

## 5. Conclusions

In summary, this study investigated the circadian rhythm of brain temperature in the acute stage of TBI and its correlation with mortality and functional outcome. Our findings suggest that circadian brain temperature rhythm in the first 72 h after surgery may be a predictor of mortality and long-term functional outcome.

## Figures and Tables

**Table 1:** Clinical parameters of patients (*n* = 108).

Parameters	Results (*n* = 108)
Male, *n* (%)	74 (68.5)
Age, years	
Mean (SD)	52.3 (20.7)
Median	52
Range	18–80
Diabetes mellitus, *n* (%)	17 (15.7)
Hypertension, *n* (%)	26 (24.1)
Diagnosis, *n* (%)	
Subdural hematoma	57 (52.8)
Epidural hematoma	15 (13.9)
Contusion	15 (13.9)
Others, including two or more	21 (19.4)
Initial Glasgow Coma Scale score	
Mean (SD)	6.9 (2.8)
Median	7
Range	3–10
ICP (24 h)	
Mean (SD)	16.4 (14.8)
CCP (24 h)	
Mean (SD)	78.5 (15.0)
12-month Glasgow Outcome Scale (%)	
1	20 (18.5)
2	19 (17.6)
3	24 (22.2)
4	23 (21.3)
5	22 (20.4)
Mean (SD)	3.1 (1.4)

SD: standard deviation; ICP: intracranial pressure; CCP; cerebral perfusion pressure.

**Table 2:** Univariate analysis for postoperative outcomes.

	Unfavorable ^1^ *n* = 63	Favorable ^1^ *n* = 45	*p ^a^*	Dead ^2^ *n* = 20	Alive ^2^ *n* = 88	*p ^a^*
Male, *n* (%)	37 (58.7)	37 (82.2)	<0.01	13 (65.0)	61 (69.3)	0.71
Age (SD)	60.0 (17.9)	41.5 (19.4)	<0.01	65.2 (15.8)	49.4 (20.5)	<0.01
DM, *n* (%)	14 (22.2)	3 (6.7)	<0.05	4 (20.0)	13 (14.8)	0.56
HTN, *n* (%)	20 (31.7)	6 (13.3)	<0.05	5 (25.0)	21 (23.9)	0.92
GCS	6.6 (2.5)	8.7 (2.7)	<0.01	6.2 (2.8)	7.7 (2.8)	<0.05
WBC > 10,000/μL, *n* (%)	42 (66.7)	36 (80.0)	0.13	15 (75.0)	63 (71.6)	0.76
Glucose > 120 mg/dL, *n* (%)	53 (84.1)	25 (55.6)	<0.01	16 (80.0)	62 (70.5)	0.39
ICP (24 h) > 20 mmHg, *n* (%)	20 (31.7)	11 (24.4)	0.41	12 (60.0)	19 (21.6)	<0.01
CPP (24 h), (SD)	77.1 (17.2)	80.5 (11.3)	0.24	63.0 (19.0)	82.1 (11.4)	<0.01
Mesor (SD)	37.2 (1.5)	37.7 (0.5)	<0.05	36.0 (2.2)	37.7 (0.5)	<0.01
Mesor 50%, *n* (%)	26 (41.3)	28 (62.2)	<0.05	4 (20.0)	50 (56.8)	<0.01
Intact rhythm of brain Temperature, *n* (%)	30 (47.6)	33 (73.3)	<0.01	8 (40.0)	55 (62.5)	0.07

SD: standard deviation; ICP: intracranial pressure; CCP: cerebral perfusion pressure; DM: diabetes mellitus; HTN: hypertension; WBC: white blood cell; GCS: Glasgow Coma Scale; ^1^: Favorable outcome was defined as GOS 4–5 at 12 months after surgery, and unfavorable outcome was defined as GOS 1–3 at 12 months after surgery; ^2^: Survival status was followed-up 12 months after surgery; *^a^*: *p*-values were assessed using a *t*-test for continuous variables and chi-square test for dichotomous variables.

**Table 3:** Univariate analysis for postoperative outcomes with sex comparison.

	Unfavorable ^1^		Favorable ^1^		Alive ^2^	
	Male *n* = 37	Female *n* = 26	Male *n* = 37	Female *n* = 8	Male *n* = 61	Female *n* = 27
Age (SD)	-	-	-	-	45.7 (20.2)	57.7 (19.0) **
DM, *n* (%)	5 (13.5)	9 (34.6) *	-	-	6 (9.8)	7 (25.9) *
Glucose > 120 mg/dL, *n* (%)	-	-	18 (48.6)	7 (87.5) *	38 (62.3)	24 (88.9) *
ICP (24 h) > 20 mmHg, *n* (%)	-	-	-	-	18 (29.5)	1 (3.7) **
Mesor mid 50%, *n* (%)	-	-	20 (54.1)	8 (100.0) *	-	-

SD: standard deviation; ICP; intracranial pressure; DM: diabetes mellitus; GCS: Glasgow Coma Scale; ^1^: Favorable outcome was defined as GOS 4–5 at 12 months after surgery, and unfavorable outcome was defined as GOS 1–3 at 12 months after surgery; ^2^: Survival status was followed-up 12 months after surgery; *: *p* < 0.05, *p*-values were assessed using a t-test for continuous variables and chi-square test for dichotomous variables; **: *p* < 0.01.

**Table 4:** Multivariate logistic regression.

	Functional Outcome ^1^ (Favorable/Unfavorable)		Survival Rate ^2^ (Alive/Dead)	
	Coefficient	OR (95% CI)	Coefficient	OR (95% CI)
Age	−0.7 **	0.94 (0.90–0.97)	−0.04*	0.96 (0.93–1.00)
GCS	0.43 **	1.53 (1.21–1.93)		
Glucose > 120 mg/dL	−1.57 *	0.21 (0.06–0.76)		
ICP (24 h) > 20 mmHg	−0.98	0.38 (0.07–2.10)		
CPP (24 h)	0.03	1.21 (0.97–1.09)		
Mesor 50%			1.56 **	4.77(1.40–16.21)
Rhythm of Brain temperature	1.67 **	5.28 (1.61–17.64)	0.73	2.07 (0.70–6.13)

OR: odds ratio; GCS: Glasgow Coma Scale; ICP: intracranial pressure; CCP: cerebral perfusion pressure; CI: confidence interval; ^1^: Favorable outcome was defined as GOS 4–5 at 6 months after surgery; unfavorable outcome was defined as GOS 1–3 at 6 months after surgery; ^2^: Survival status was followed-up at 12 months after surgery; * *p* < 0.05; **: *p* < 0.01.

## Data Availability

All data relevant to the study are included in the article.
